# The G Protein-Coupled Receptor RAI3 Is an Independent Prognostic Factor for Pancreatic Cancer Survival and Regulates Proliferation via STAT3 Phosphorylation

**DOI:** 10.1371/journal.pone.0170390

**Published:** 2017-01-23

**Authors:** Elisabeth Jahny, Hai Yang, Bin Liu, Beatrix Jahnke, Franziska Lademann, Thomas Knösel, Petra Rümmele, Robert Grützmann, Daniela E. Aust, Christian Pilarsky, Axel Denz

**Affiliations:** 1 Department of Surgery, TU Dresden, Fetscherstraße 74, Dresden, Germany; 2 Department of Surgery, Universitätsklinikum Erlangen, Krankenhausstraße 12, Erlangen, Germany; 3 Institute of Pathology, Ludwig-Maximilians-Universität München, München, Germany; 4 Institute of Pathology, Universitätsklinikum Erlangen, Krankenhausstraße 8–10, Erlangen, Germany; 5 Institute of Pathology, TU Dresden, Fetscherstraße 74, Dresden, Germany; Vrije Universiteit Brussel, BELGIUM

## Abstract

Pancreatic Ductal Adenocarcinoma (PDAC) is one of the deadliest tumors worldwide. Understanding the function of gene expression alterations is a prerequisite for developing new strategies in diagnostic and therapy. GPRC5A (RAI3), coding for a seven transmembrane G protein-coupled receptor is known to be overexpressed in pancreatic cancer and might be an interesting candidate for therapeutic intervention. Expression levels of RAI3 were compared using a tissue microarray of 435 resected patients with pancreatic cancer as well as 209 samples from chronic pancreatitis (CP), intra-ductal papillary mucinous neoplasm (IPMN) and normal pancreatic tissue. To elucidate the function of RAI3 overexpression, siRNA based knock-down was used and transfected cells were analyzed using proliferation and migration assays. Pancreatic cancer patients showed a statistically significant overexpression of RAI3 in comparison to normal and chronic pancreatitis tissue. Especially the loss of apical RAI3 expression represents an independent prognostic parameter for overall survival of patients with pancreatic cancer. Suppression of GPRC5a results in decreased cell growth, proliferation and migration in pancreatic cancer cell lines via a STAT3 modulated pathway, independent from ERK activation.

## Introduction

Pancreatic cancer is one of the most perilous and lethal solid malignancies and the five-year relative survival rate, is one of the lowest for all cancers with 8% [1]. Typical alterations in the molecular signaling include mutations in KRAS in 95% of PDACs and in tumor suppressor’s genes like p16/CDKN2A, p53 or SMAD4 [2]. Besides these, many other changes in different pathways have been described, which are caused by gene mutations as well as epigenetic mechanisms [2, 3].

The G-protein-coupled receptor family C, member 5, group A (GPRC5A); or retinoic acid-inducible 3 (RAI3) gene was firstly described in 1998 as a seven transmembrane helices protein with a molecular weight of 40 kDa and its expression is regulated by all-trans-retinoic acid (ATRA) [4]. GPRC5a is over expressed in pancreatic, breast, gastric and colon cancer, and it has been shown that overexpression of RAI3 predict poor prognosis in hepatocellular carcinoma [5–11]. Functional analysis of GPRC5a in different carcinomas indicated that RAI3 has pleiotropic effects and might confer resistance to Gemcitabine in pancreatic cancer, however the ligand of GPRC5a remains elusive [12], but GPRC5a overexpression might lead to a reduction of EGFR levels [13–16].

In this study, we investigated the expression and function of RAI3, in pancreatic cancer. We verified the reported RAI3 RNA over-expression using immunohistochemistry and we could show that apical RAI3 expression is an independent prognostic marker for overall survival. The siRNA mediated knock-down of RAI3 lead to proliferation and migration inhibition via STAT3 inactivation.

## Material and Methods

### Tissue microarray (TMA): patient samples and immunohistochemistry

Under consideration of the appropriate guidelines, TMA were prepared and evaluated [17]. The tissue was collected from routine surgery dissections and the histological diagnosis was confirmed by an expert histopathologist. Samples of 598 patients were collected with approval by the ethics committee of the Technical University Dresden, University Jena and University Regensburg for biobanking purposes and written informed consent of each patient was obtained. Records of the informed consent are part of the biobank database. The use of the tissue for TMA construction and immunohistochemical staining was approved by the ethics committee.

Immunohistochemical staining was performed with a VENTANA BenchMark XT (Ventana, Roche Diagnostics, Freiburg) system. For deparaffinization and retrieval of antigen, the slides were heated and pretreated with CC1 (Ventana). Subsequently, peroxidase was blocked and the incubation with RAI3 antibody (1:400, Novus Biologicals, Wiesbaden, NBP1-89743) followed. The detection was performed using UltraView Universal DAB Detection Kit (Ventana). Finally, the slides were counterstained with haematoxilin and Bluing Reagent (Ventana). As a negative control rabbit IgG was used. The scoring of the TMA was performed by two scientists (EJ, DA), independent on clinical data. Three parameters were pre-defined: the intensity of the staining (ordinal scale, 0–3 points), the portion of stained ductal cells (metric scale, 0–100%, called “expression”) and the portion of strictly apical stained cells as part of all stained ductal cells (metric scale, 0–100%, called “apical expression”). Patients were only included in the evaluation, when minimum one TMA core was interpretable. For a TMA core with an intensity of 0 points the expression is assessed with 0% and the apical expression is not assessable. The cut-off of >30% of apical stained cells were used in the univariate analysis.

Samples of 501 patients (83.8% of the samples) could be included in the analysis of RAI3-expression. Of these were 435 of pancreatic adenocarcinoma (tumor samples), 34 of Intraductal Papillary Mucinous Neoplasm (IPMN) and 32 of chronic pancreatitis. Normal ductal tissue adjacent to the tumor was used for comparison in 143 samples.

Analysis of clinico-pathological and survival data were performed for 376 patients with pancreatic adenocarcinoma and a postoperative survival time over 30 days.

### Cell culture and transfection with small interfering RNAs

Pancreatic carcinoma cell lines MiaPaCa-2 (CRL-1420), Panc1 (CRL-1469) and AsPc-1 (CRL-1682) were purchased from ATCC and grown in monolayer culture in a humidified atmosphere containing 5% CO_2_ at 37°C. The culture medium for MiaPaCa-2 consists of DMEM+GlutaMax with high glucose (Thermo Fisher Scientific, Braunschweig, Germany) supplemented by 10% fetal bovine serum (Sigma-Aldrich, Taufkirchen, Germany) and 2.5% horse serum (Thermo Fisher Scientific). Panc1-cells were cultured in RPMI 1640 (Thermo Fisher Scientific) with 10% fetal bovine serum (Sigma-Aldrich). For culturing AsPc-1 cells, RPMI 1640 (Thermo Fisher Scientific) were used, complemented by 10% fetal bovine serum (Sigma-Aldrich), 10 mM Hepes (Thermo Fisher Scientific), 1 mM sodium pyruvate (Thermo Fisher Scientific) and 4,5 g/l glucose (B. Braun Melsungen AG, Melsungen, Germany). Primary cell lines were isolated and cultivated as described by Rückert et al. [18].

The transfection with siRNA followed the protocol of Harborth et al [19]. Two different negative controls were used, medium containing the transfection reagent (NC1) and the non-silencing siRNA allstars (NC2; #SI03650318 Qiagen Hilden, Germany). The siRNA Eg5 [19] positive control and the GPRC5a-siRNA (GPRC5A.1: 5’-CAACUCAAGUUUGAGCCCUUA(dTdT)-3’; GPRC5A.6: CAGGATGTTATCGCTATTGAA) were ordered from Eurofins MWG Operon (Ebersberg, Germany) and Qiagen respectively. Transfection was performed using Oligofectamine (Thermo Fisher Scientific) with 5 x 10^4^ cells per well, seeded one day before the transfection, and 68,7 nM siRNA. The result of the RNA interference experiment was evaluated after 48 or 72 hours. The determination of the life cell count was performed with a counting chamber (Neubauer; Marienfeld, Germany) or an automated cell counter (BIO-RAD, Munich, Germany).

### Functional assays

As a assay of seeding efficiency after 72 hours of siRNA transfection cells were detached and 3.000 cells were seeded in 12-well-plates. Cells grow for seven days and stained with Coomassie Brilliant Blue staining solution (Brillant Blau G 250, Roth, Karlsruhe, Germany). Quantification was performed by Gene Tools Analysis software (Syngene, Cambridge, UK).

After 48 h of transfection cells were seeded in Fluoro Blok Individual Cell Inserts (Becton Dickinson, Heidelberg, Germany) with serum-free medium. Inserts were transferred in 24-Well-plates containing complete growth medium with 20% fetal bovine serum. The cells were allowed to migrate in a period of exactly 16 hours. The inserts were washed in PBS (Thermo Fisher Scientific), fixed with Methanol and stained with DAPI (Sigma-Aldrich). The distribution of migrated cells was assessed, four image sections per well photographed and counted with Image J (Wayne Rasband, National Institutes of Health).

### Quantitative RT-PCR and western blot

The isolation of RNA was performed with miRNeasy® Mini Kit (Qiagen) and the cDNA was synthesized with High Capacity cDNA Reverse Transcription Kit (Thermo Fisher Scientific) The quantitative RT-PCR was performed with Power SYBR® Green PCR Master Mix (Thermo Fisher Scientific) and analyzed by 2^-ΔΔCt^ method [20]. The housekeeping gene β-Actin was used as reference-value. Primer sequences for amplification were β-Actin-F (5’-AAATCTGGCACCACACCTTC-3’), β-Actin-R (5’-AGAGGCGTACAGGGATAGCA-3’), GPRC5a-F (5’-TCTATGCCCCCTATTCCACA-3’) and GPRC5a-R (5’-TGCATTTGTCCCACTCTTCA-3’) (Eurofins MWG Operon).

Cells were lysed with RIPA-buffer, gel electrophoresis was performed under reducing conditions with acrylamide gels (NuPAGE Novex 4–12% Bis-Tris Gele, Thermo Fisher Scientific) and proteins were transferred to a nitrocellulose membrane (GE Healthcare Life Science, Freiburg, Germany). As primary antibodies RAI3 (1:10,000, Novus Biologicals, NBP1-89743), ERK (1:1,000, Cell Signaling #9102), phospho-ERK (1:1,000, Cell Signaling #9101) STAT3 (1:1,000, Cell Signaling #9139) and phospho-STAT3 Tyr705 (1:1,000, Cell Signaling #9145) were used for detection. The antibodies α-Tubulin (1:5,000, Sigma-Aldrich, T9026) and GAPDH (1:1,000, Cell Signaling, #2118) served as loading controls. Signal detection was performed using Immobilon^TM^ Western Chemilumineszenz HRP Substrate (Merck Chemicals, Darmstadt, Germany) and HRP-linked anti-rabbit IgG (1:1,000, Cell Signaling, #7074) or anti-mouse IgG (1:1,000, Cell Signaling, #7076). Quantification of signals was performed by the Gene Tools Analysis software (Syngene, Cambridge, UK).

### Statistical analysis

Statistical analysis was performed with IBM SPSS® Statistics Version 22 and Microsoft Excel® Version 2013. For cell culture experiments t-test was used. Within the evaluation of the immunohistochemistry Kruskal-Wallis test, Fisher’s exact test, Mann-Whitney test, Receiver Operating Characteristic (ROC) curve, Kaplan-Meier curve with Log-Rank test for univariate analyses as well as a Cox regression for multivariate analysis was applied. For interpretation of differences a p-value ≤ 0.05 was considered as statistically significant.

## Results

**Fig 1:**
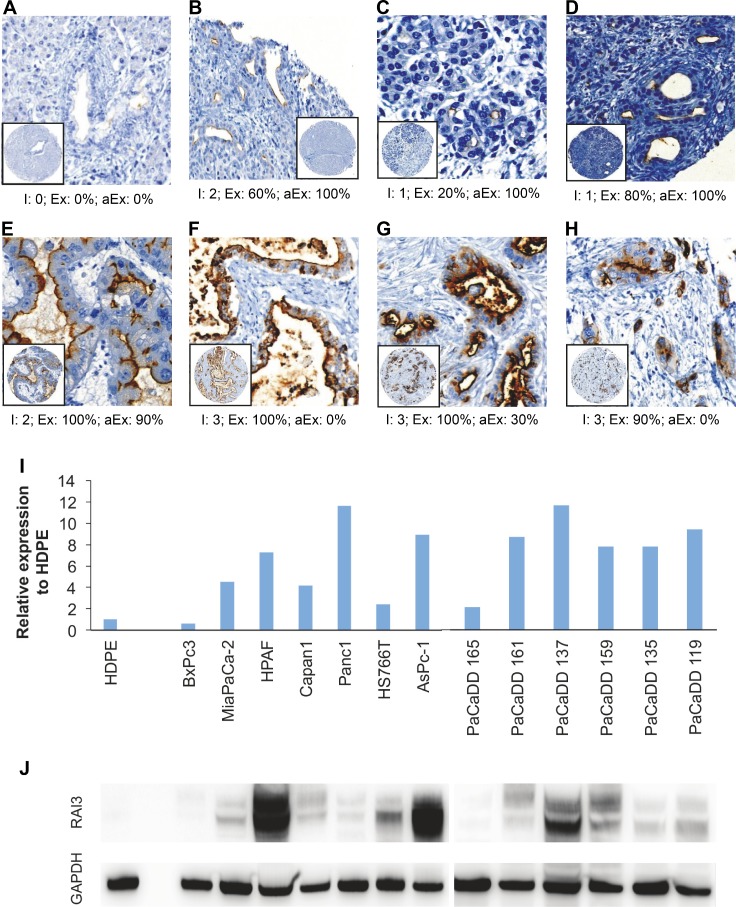
Expression of RAI3 in human tissue and cell lines. TMA—Immunochemistry with RAI3-Antibody in different types of pancreatic tissue. Presentation in 20-fold magnification of A-C: Normal; D: Chronic pancreatits (CP), E: Intraductal Papillary Mucinous Neoplasm (IPMN); F—H: Pancreatic Ductal Adenocarcinoma (PDAC). Three parameters were considered: RAI3-Intensity (I), RAI3-Expression (Ex) and Apical RAI3-Expression (aEx); I,J: Differential expression of RAI3 mRNA respective protein in common pancreatic cell lines and primary pancreatic cell lines measured by qRT-PCR (I) and Western Blot (J) with a conspicuously low expression in BxPc3 and PaCaDD165 in comparison to the other cell lines in the group. (n = 3).

**Fig 2:**
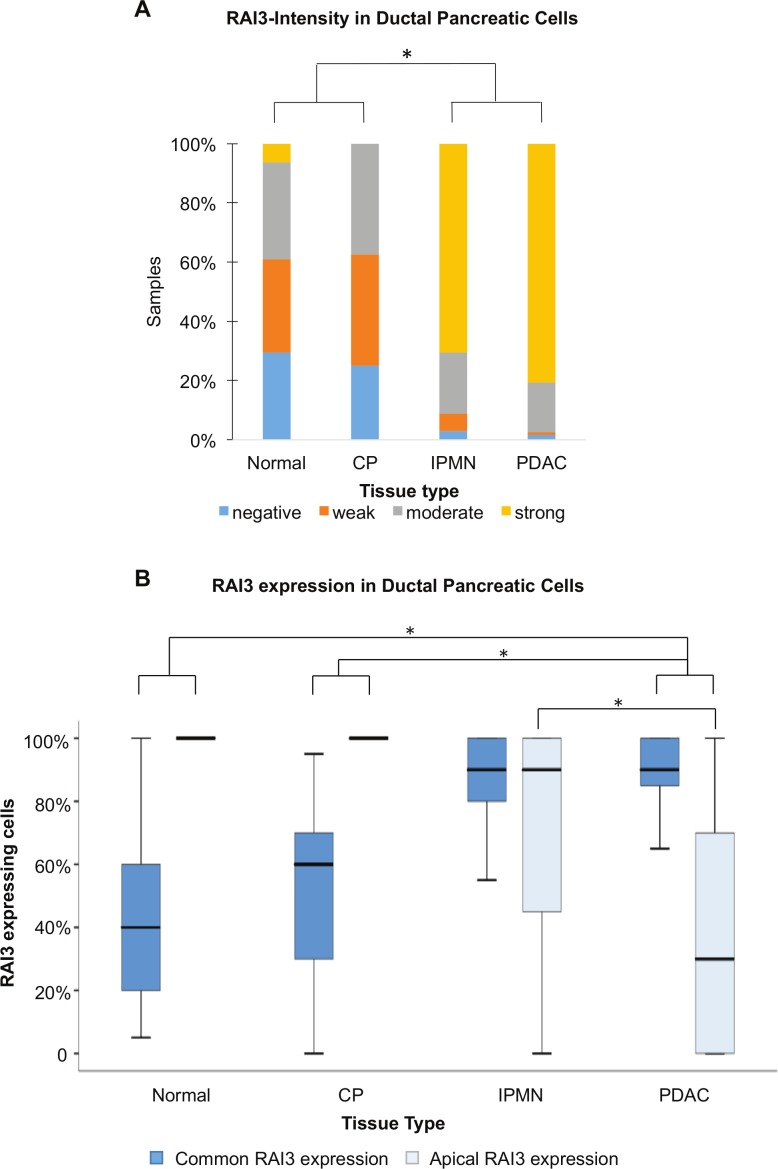
Intensity and Expression of RAI3 in Ductal Pancreatic Cells of different tissue types. A: Relative distribution of RAI3-Intensity (* indicates statistically significant differences (p < 0.001) according to Fisher‘s exact test); B: Comparison of the parameters Common Rai3-Expression and Apical Rai3-Expression (* indicates statistically significant differences (p < 0.001) according to Mann-Whitney-Test with Bonferroni-adjustment).

In PDAC and IPMN the percentage of RAI3 expressing cells is highly elevated (median = 90%) compared to normal (median = 40%) and CP tissue (median = 60%) and PDAC cells show mostly a strong expression. Apical expression was detected in 100% of stained normal and CP tissue as well as in 90% of the stained IPMNs. PDAC-tissue showed an obvious loss of apical expression (median = 30%) (Fig 1A–1F and Fig 2A and 2B) (Kruskal-Wallis-test p<0.001 for all parameters).

**Fig 3:**
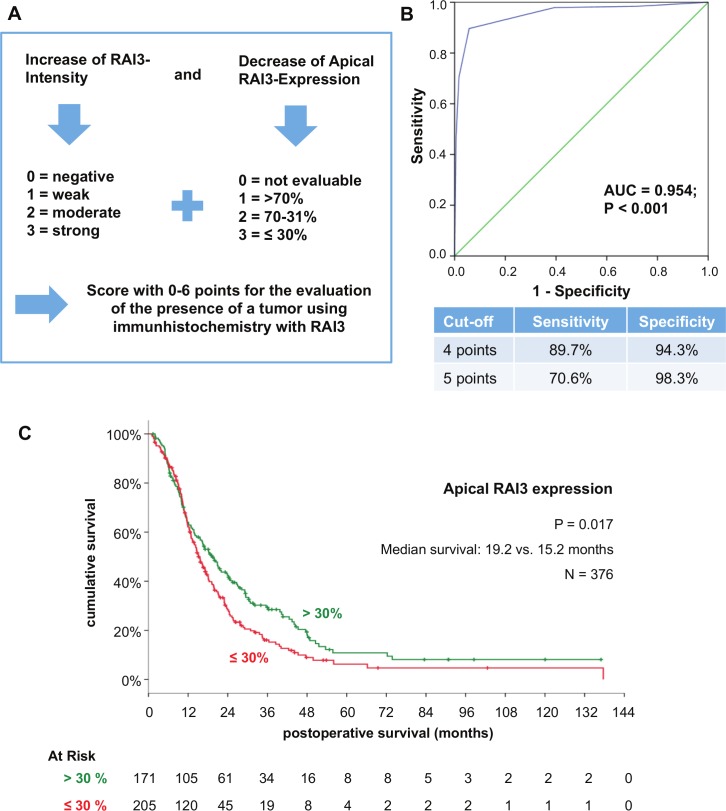
RAI3 immunohistochemistry as diagnostic and prognostic instrument. A: Definition and calculation of a diagnostic score for the presence of a pancreatic ductal adenocarcinoma (PDAC); B: Receiver Operating Characteristic (ROC) curve for the diagnostic score using for distinction of PDAC from Normal or Chronic Pancreatitis (CP) tissue (Sensitivity and specificity were calculated for two exemplary cut-off-points); C: Univariate Kaplan-Meier analysis (statistically significant differences between apical expression over 30% and less (p = 0.017) according to Log-Rank-test).

A diagnostic score to differentiate pancreatic cancer from CP or normal tissue, was calculated by addition of increasing RAI3-intensity and decreasing apical RAI3-expression displayed an AUC of 0.954 (p<0.001). Assuming that a value of minimum 4 (respective 5) of 6 points is typically for tumorous tissue, the diagnostic score exhibits a calculated sensitivity of 89.7% (respective 70.6%) and specificity of 94.3% (respective 98.3%) (Fig 3A and 3B).

Univariate analysis the clinic-pathological data of the 376 patients with PDAC analyzed displayed a significant difference (p<0.05) in survival concerning the pN-stage (regional lymph nodes affected, histology grade, resection margin and apical RAI3 expression (Table 1). Patients with an apical RAI3-Expression of 30% or lower showed a loss of 4 months in median survival time (p = 0.017, Fig 3C). Cox-regression analysis revealed that apical RAI3-expression is a independent prognostic factor for the survival of the patients (Table 2, RR: 1.37; p < 0.05).

**Table 1:** Univariate analysis of clinico pathological variables of the 376 patients with pancreatic cancer analyzed on the TMA.

Variables		N		%	OS (months)	p-Value (Log-Rank)
**Total cohort**		376		100	16.49	
**Age (at OP-time)**						
	Mean (years)	64.0 ± 0.51				
	Median (years)	66				
	Range (years)	33–84				
	to 65 years	176		46.8	18.10	0.120
	over 65 years	200		53.2	15.11	
**Gender**						
	male	202		53.7	17.15	0.791
	female	174		46.3	15.18	
**Origin**						
	Jena	96		25.5	18.10	0.962
	Regensburg	81		21.5	14.55	
	Dresden	199		52.9	16.49	
**T stage**						
	T1/2	57		15.2	19.65	0.875
	T3/4	319		84.8	16.13	
**N stage**						
	negative	115		30.6	22.93	**0.005**
	positive	261		69.4	15.60	
**M stage**						
	negative	301		80.0	17.28	0.236
	positive	15		4.0	11.66	
	unknown	60		16.0	-	
**Histology grade**						
	G1/2	179		47.6	21.03	**<0.001**
	G3/4	197		52.4	13.93	
**Resection margin**						
	R0	277		73.7	19.15	**0.007**
	R1/2	99		26.3	12.88	
**Adjuvant therapy**						
	no	252		67.0	15.70	0.085
	yes	124		33.0	19.88	
**RAI3-Intensity**						
	negative to moderate	66		17.6	19.15	0.926
	strong	310		82.4	16.23	
**RAI3-Expression**						
	< 90%	106		28.2	15.60	0.338
	≥ 90%	270		71.8	16.79	
**Apical RAI3-Expression**						
	> 30%	171		44.8	19.15	**0.017**
	≤ 30%	205		53.7	15.18	

Presentation of the investigated 376 patients with pancreatic adenocarcinoma, including their clinicopathological data and the results of univariate analysis. P-values are calculated by Log-Rank-test and statistically significant differences (p<0.05) in overall survival (OS) subsequently marked by bold p-values.

**Table 2:** Multivariate Cox proportional hazard model for different variables.

	Variables	Hazard Ratio	95% CI	Significance
**Step 1**				
	Histology Grade (high grade vs. low grade)	1.514	1.172–1.957	0.002
**Step 2**				
	Histology Grade (high grade vs. low grade)	1.570	1.211–2.036	0.001
	Adjuvant Therapy (yes vs. no)	0.753	0.574–0.987	0.040
**Step 3**				
	N-stage (positive vs. negative)	1.382	1.039–1.838	0.026
	Histology Grade (high grade vs. low grade)	1.501	1.154–1.952	0.002
	Adjuvant Therapy (yes vs. no)	0.727	0.553–0.954	0.022
**Step 4**				
	N-stage (positive vs. negative)	1.447	1.084–1.931	0.012
	Histology Grade (high grade vs. low grade)	1.354	1.027–1.784	0.032
	Adjuvant Therapy (yes vs. no)	0.705	0.537–0.928	0.012
	Apical RAI3-Expression (≤30% vs. >30%)	1.371	1.040–1.806	0.025

Presentation of the results of multivariate analysis by cox regression. Statistically significant independent parameters for overall survival are N-Stage, Histology Grade, Adjuvant Therapy and Apical RAI3-Expression.

To elucidate the role of RAI3 further we first compared expression levels in pancreatic cancer cell lines by Western blot and quantitative RT-PCR. The immortalized human ductal pancreatic epithelial (HDPE) cell line [21] showed only negligible expression of RAI3 protein or GPRC5A mRNA expression, whereas pancreatic cancer lines displayed highly variable but detectable over-expression (Fig 1G and 1H).

**Fig 4:**
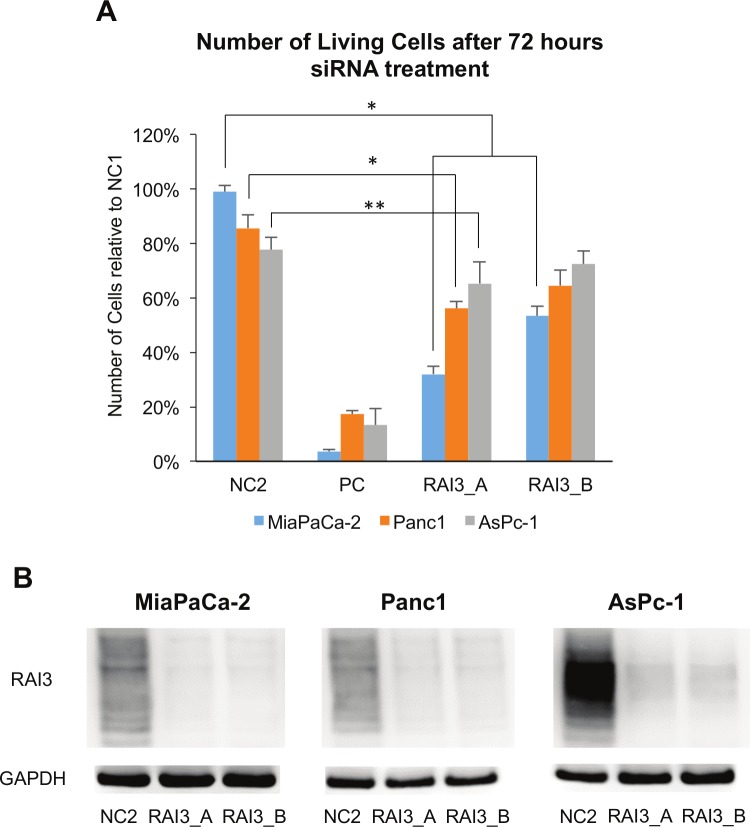
Effect of treatment with GPRC5a-siRNA over 72 hours. A: Number of living cells in negative controls (NC; 1: Medium, 2: non-sense-siRNA), positive control (PC; eg5-siRNA) and knock-down with two different GPRC5a-siRNA (RAI3_A and RAI3_B). Statistically significant differences in t-test are marked with * for P-Value < 0.001 and ** for P-Value < 0.05; B: Western blot and quantitative real time PCR with RAI3 antibody confirms the successful knock-down using siRNA (for functional analysis the GPRC5A.1 siRNA was chosen) (S1 Fig). (n = 5).

**Fig 5:**
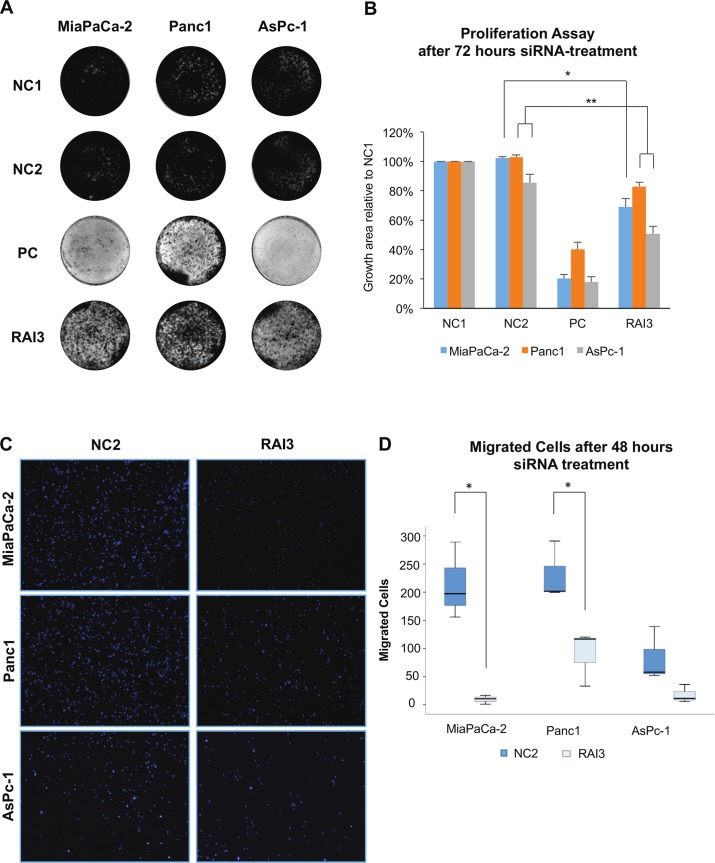
Effect of siRNA based knock-down on the colony formation and migration of pancreatic cancer cell lines. A: Examples of the results of typical colony forming assays over 7 days after siRNA-treatment; B: Growth area compared to NC1 (statistically significant difference between RAI3 and NC2 is demonstrated by * for P<0.01 and ** for P<0.001 performed by t-test); C: Typical images of migrated cells in negative control (NC) and knock-down with GPRC5a siRNA (RAI3) in different cell lines; D: Number of migrated cells after 48 hour siRNA-treatment and subsequently 16 hours migration assay (* indicates statistically significant differences (P<0.05) in t-test between the negative control with non-sense-siRNA (NC) and the knock-down with GPRC5a-siRNA (RAI3)). (n = 3).

In siRNA based knock-down experiments both GPRC5A-siRNAs led to a significant decrease in cell number in Panc1, MiaPaCa-2 cells and AsPc-1 cells (P<0.05) compared to the negative control (Fig 4A) and were able to suppress RAI3 expression (Fig 4B). Moreover, colony forming was impaired in siRNA treated cells in MiaPaCa-2, Panc1 and AsPc-1 cells (P<0.01). RAI3 suppression inhibited colony forming in MiaPaCa-2 cells by 69%, in Panc1 by 83% and in AsPc-1 by 51% (Fig 5A and 5B). Furthermore, the treatment with the GPRC5a-siRNA showed a large effect on the migratory ability of the cells, since only 5.0% [±2.5%] of MiaPaCa-2, 38.9% [±10.3%] of Panc1 and 31.0% [±16.0%] of AsPc-1 migrated compared to the control (Fig 5C and 5D).

**Fig 6:**
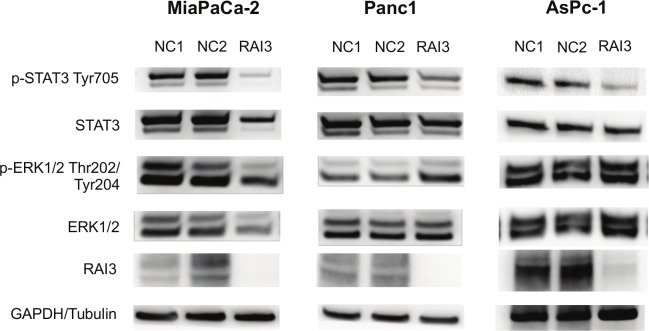
Western Blot analysis of different pathways were tested for alteration in knock-down with GPRC5A siRNA in comparison to negative control. Phosphorylation of STAT3 at Tyr705 is reduced in knock-down of all three cell lines, whereas Phospho-ERK is not changed significantly (see S2 Fig for original Western blot images). (n = 3).

A central pathway that influences the cell proliferation and migration is the Ras/Raf/MEK/ERK pathway. Analysis of the proteins ERK 1/2 and their activating phosphorylation sides phospho-ERK 1/2 (Thr202/Thr204) showed no alteration, but RAI3 mRNA suppression resulted in a reduced level of the phosphorylated form (Tyr705) of STAT3 in all cell lines analyzed (Fig 6).

## Discussion

The results in TMA show clearly, that RAI3/GPRC5a is over-expressed in PDAC in comparison to normal pancreas or CP tissue. Interestingly, in IPMN, a cystic neoplasm that is considered to be a precursor lesion for pancreatic cancer, also high intensity and expression levels of RAI3 were observed. Compared to other tissues the loss of apical staining in PDACs was revealed as an independent parameter for the overall survival of patients with pancreatic carcinoma. A possible background for this is the increasing loss of cell polarity and architecture with the dedifferentiation of the tumors. This could be detected earlier by immunohistochemistry than by conventional methods as seen with other marker [22].

The overexpression of RAI3 in pancreatic cancer could be confirmed in the comparison of mRNA and protein-levels in different cell lines. Thereby, RAI3 expression seems to seems to suggest a connection with the phenotypes respectively genotypes of the cell lines. The two cell lines HPAF and AsPc-1, which have a high RAI3 protein level, were isolated from ascites [23]. BxPc3 and PaCaDD 165, which have a low GPRC5A mRNA and protein level, show both a KRAS wild type [24] indicating that RAI3 expression increases with malignancy of a tumor. A previous described connection between the expression of RAI3 and an alteration of p53 in breast carcinoma cell lines [25] could not be confirmed, since cell lines with a high GPRC5A mRNA expression like PaCaDD 161 are p53 wild type and cell lines with a p53 mutation like BxPC3 display low GPRC5A level. However, the regulation of RAI3 might be dependent on the type of p53 mutation and might be co regulated by other factors like proteins of the retinoic acid receptor family [4, 26].

Our results of RNA-interference experiments indicates, that GPRC5a has some function in promoting cell growth, proliferation and migration by reducing the crucial phosphorylation of STAT3 at the position Tyr705, whereas the KRAS/MAPK signaling remains mostly unchanged.

This is consistent with the results of previous published experiments with the breast cancer cell lines T47D and MCF7, which showed a growth-inhibiting effect of GPRC5A-siRNA [14].

An alteration in proliferation ability might also influence the migration ability of the cells and could cause an ostensible effect on migration. However, in Panc1-cells, we demonstrated a high effect on migration ability, whereby the influences on proliferation were still low. Therefore, we assume a proliferation-independent effect of GPRC5A knock-down on migration ability of the PDAC-cells, which is dependent on the cellular context.

Nevertheless, we could demonstrate high overexpression of GPRC5a in PDACs. Our data lead to the suggestion, that RAI3 is an epigenetic regulated gene PDACs with oncogenic effects, which could be used for diagnostic and prognostic aspects. Since, GPRC5a is located in cell membrane; it represents a potential therapeutic target.

## Supporting Information

S1 Fig Results of GPRC5a knockdown in three pancreatic cancer cell lines measured by quantitative real time PCR from total RNA. (PDF)

S2 Fig Original Western blot images for the analysis of pathway perturbation. (ZIP)

## Abbreviations

PDAC: pancreatic ductal adenocarcinoma
CP: chronic pancreatitis
IPMN: intra-ductal papillary mucinous neoplasm
RAI3: retinoic acid induced 3
ROC: receiver operating characteristic
