# Supplementary figures and images for: The G Protein-Coupled Receptor RAI3 Is an Independent Prognostic Factor for Pancreatic Cancer Survival and Regulates Proliferation via STAT3 Phosphorylation
Source: PLoS One. 2017 Jan 23;12(1):e0170390. doi: 10.1371/journal.pone.0170390 (PMC5256936; doi:10.1371/journal.pone.0170390)

qRT-PCR

### RNA-Expressionlevel of RAI 3 after RNAi of RAI 3

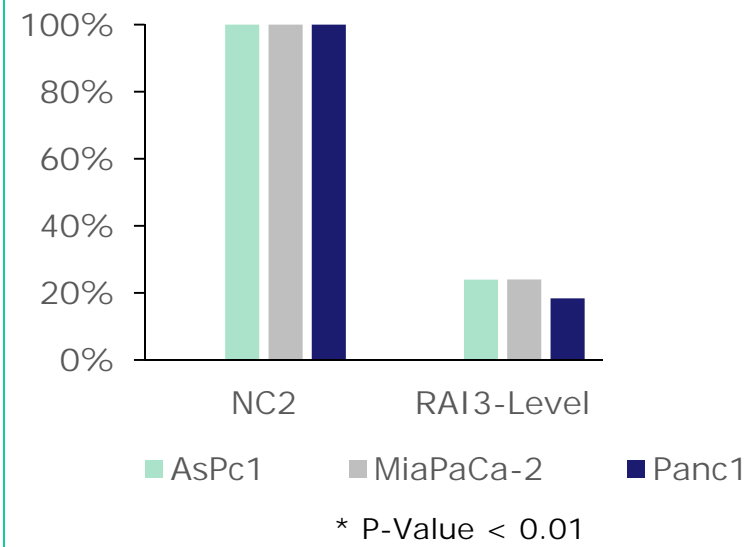

Supplement: S1 Fig — (PDF) [file pone.0170390.s001.pdf]

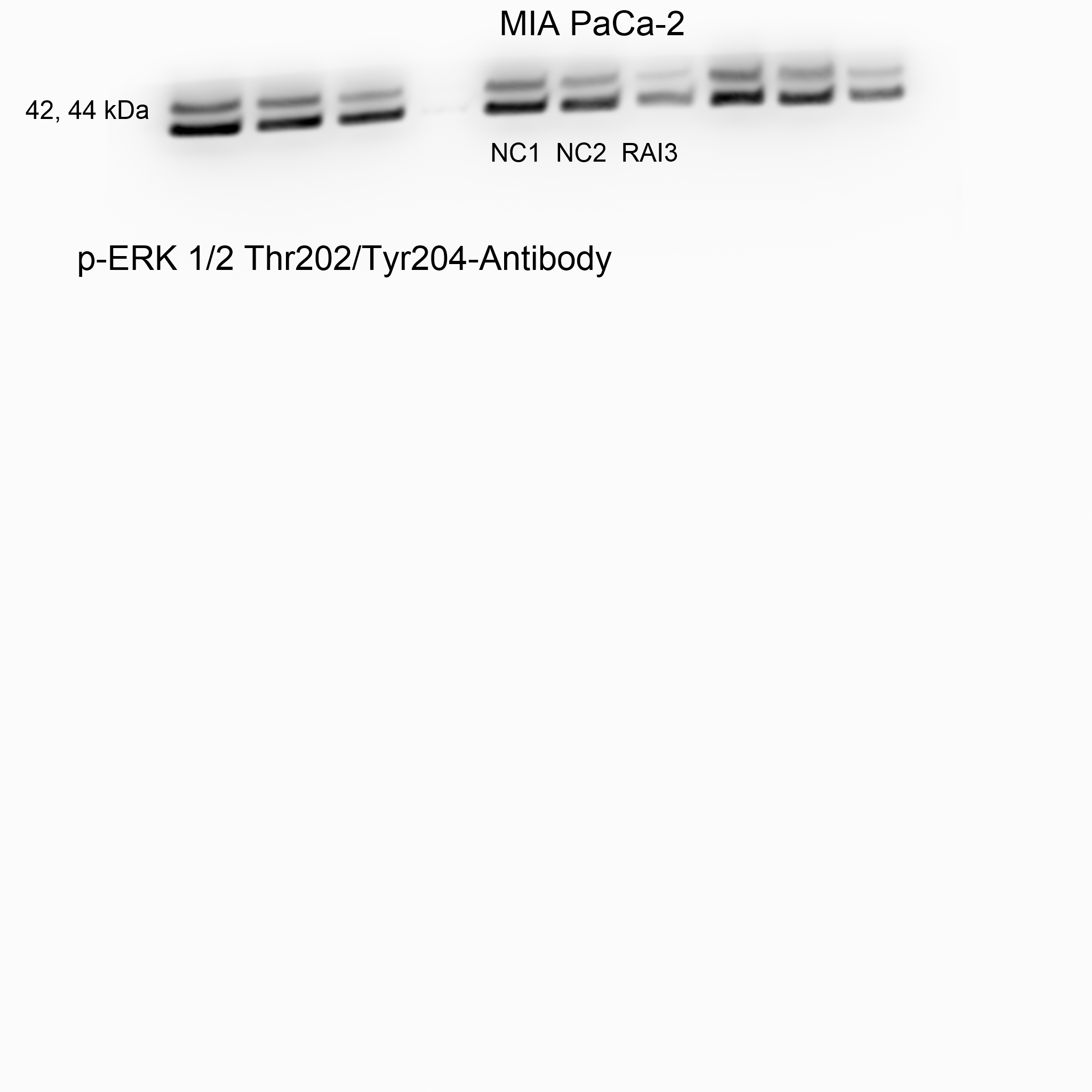

Supplement: S2 Fig — (ZIP) [file pone.0170390.s002.zip › Figure6_pERK_antibody_MIA PaCa-2.tif]

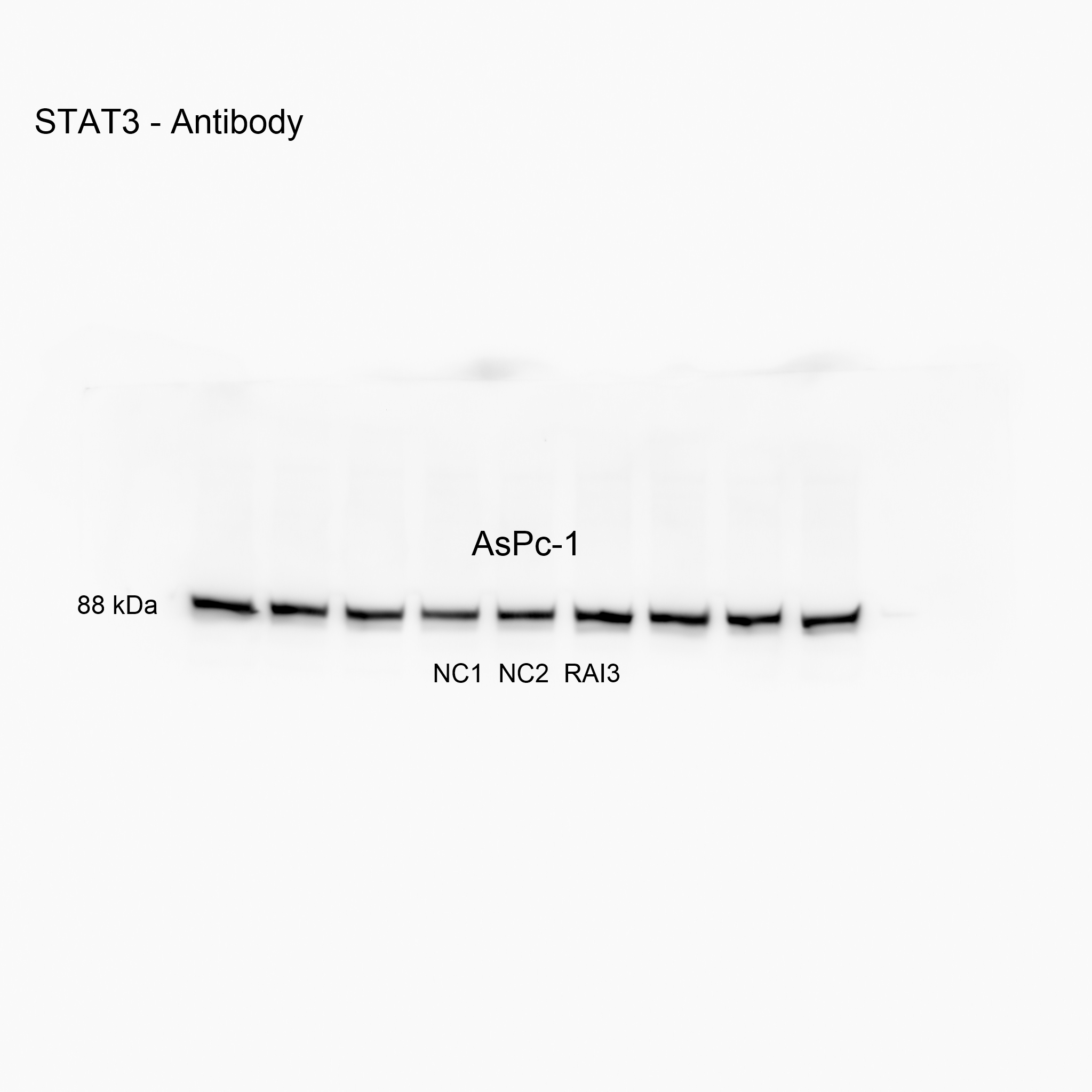

Supplement: S2 Fig — (ZIP) [file pone.0170390.s002.zip › Figure6_STAT3_antibody_AsPc-1.tif]

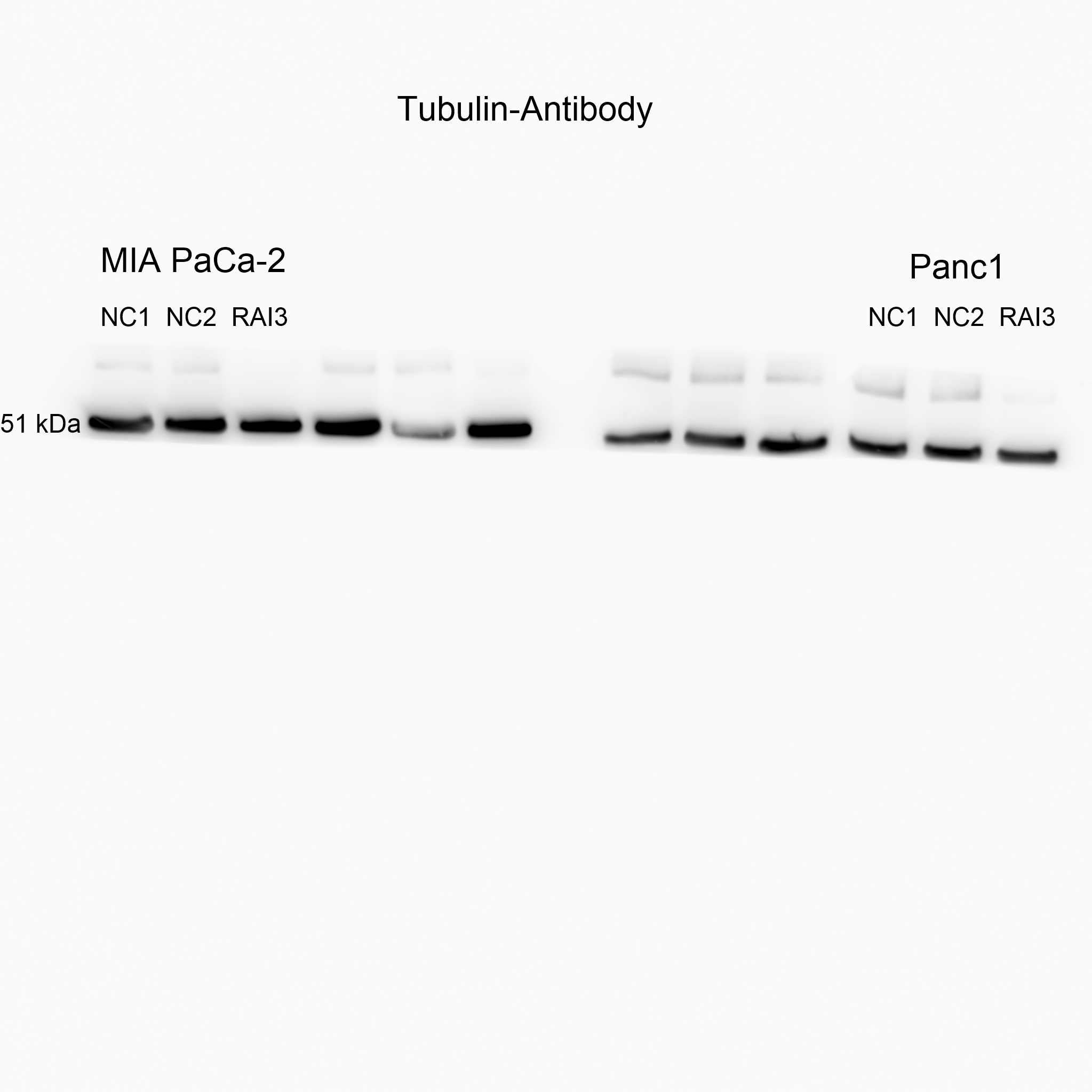

Supplement: S2 Fig — (ZIP) [file pone.0170390.s002.zip › Figure6_Tubulin_antibody_MIA_Panc1.tif]

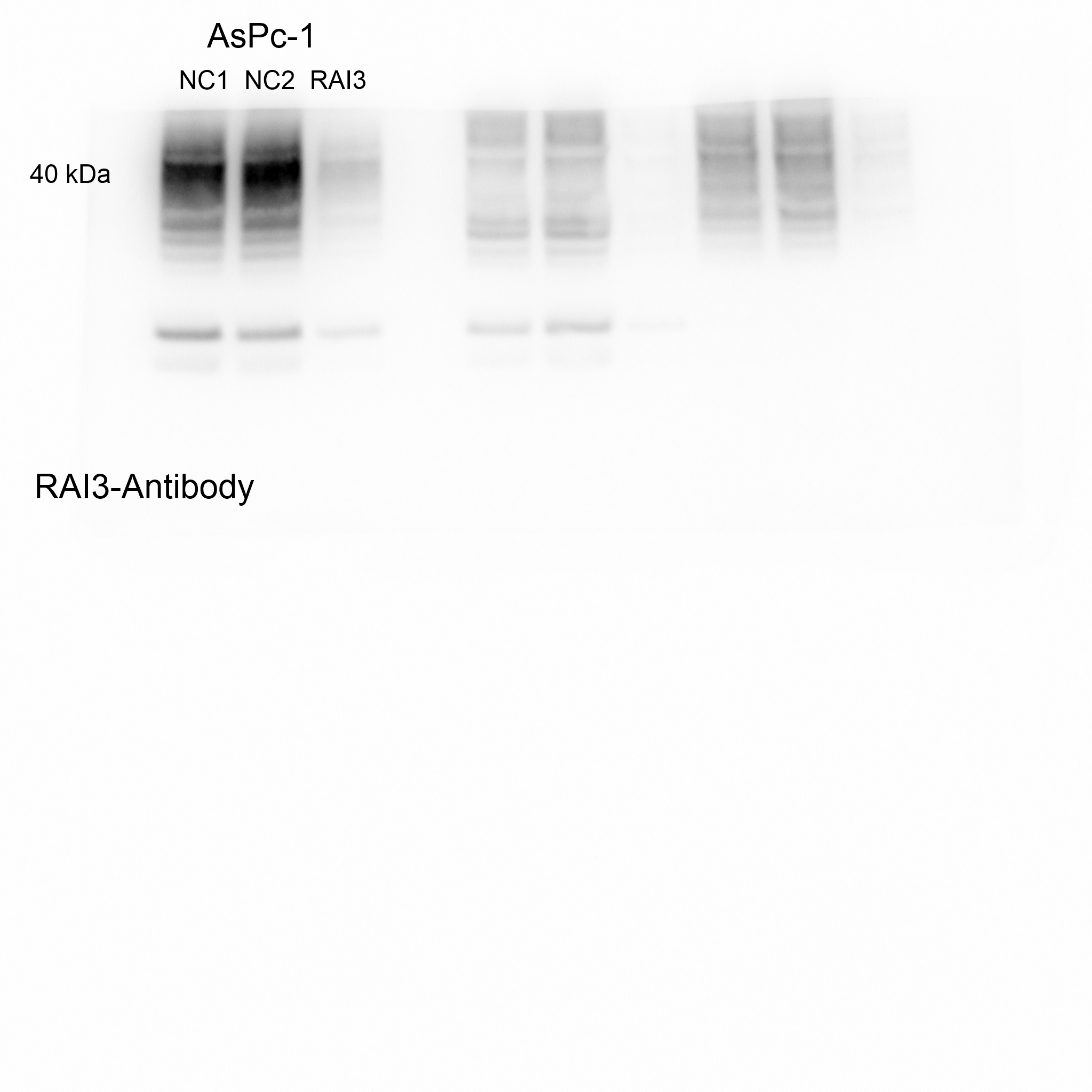

Supplement: S2 Fig — (ZIP) [file pone.0170390.s002.zip › Figure6_RAI3_antibody_AsPc-1.tif]

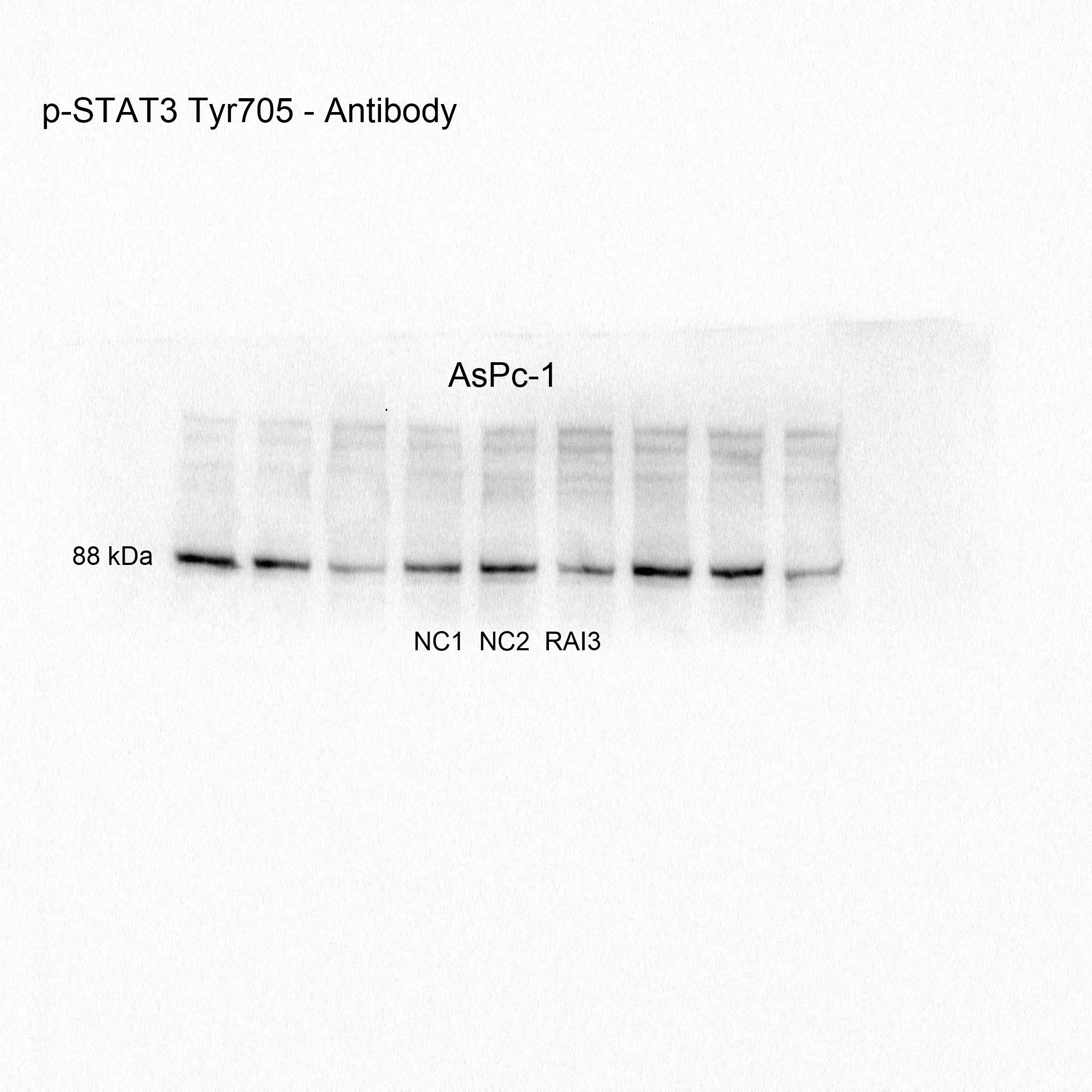

Supplement: S2 Fig — (ZIP) [file pone.0170390.s002.zip › Figure6_pSTAT3_antibody_AsPc-1.tif]

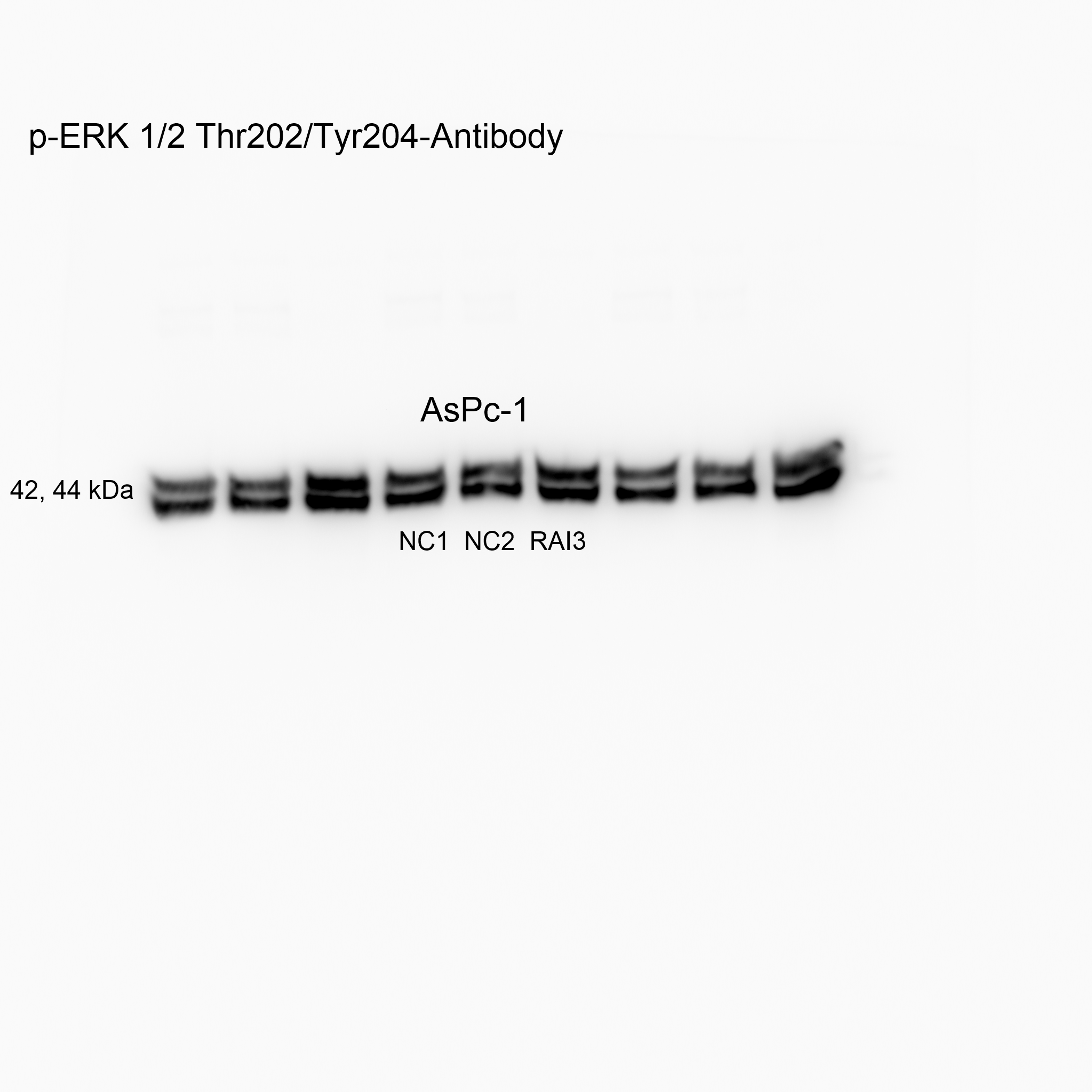

Supplement: S2 Fig — (ZIP) [file pone.0170390.s002.zip › Figure6_pERK_antibody_AsPc-1.tif]

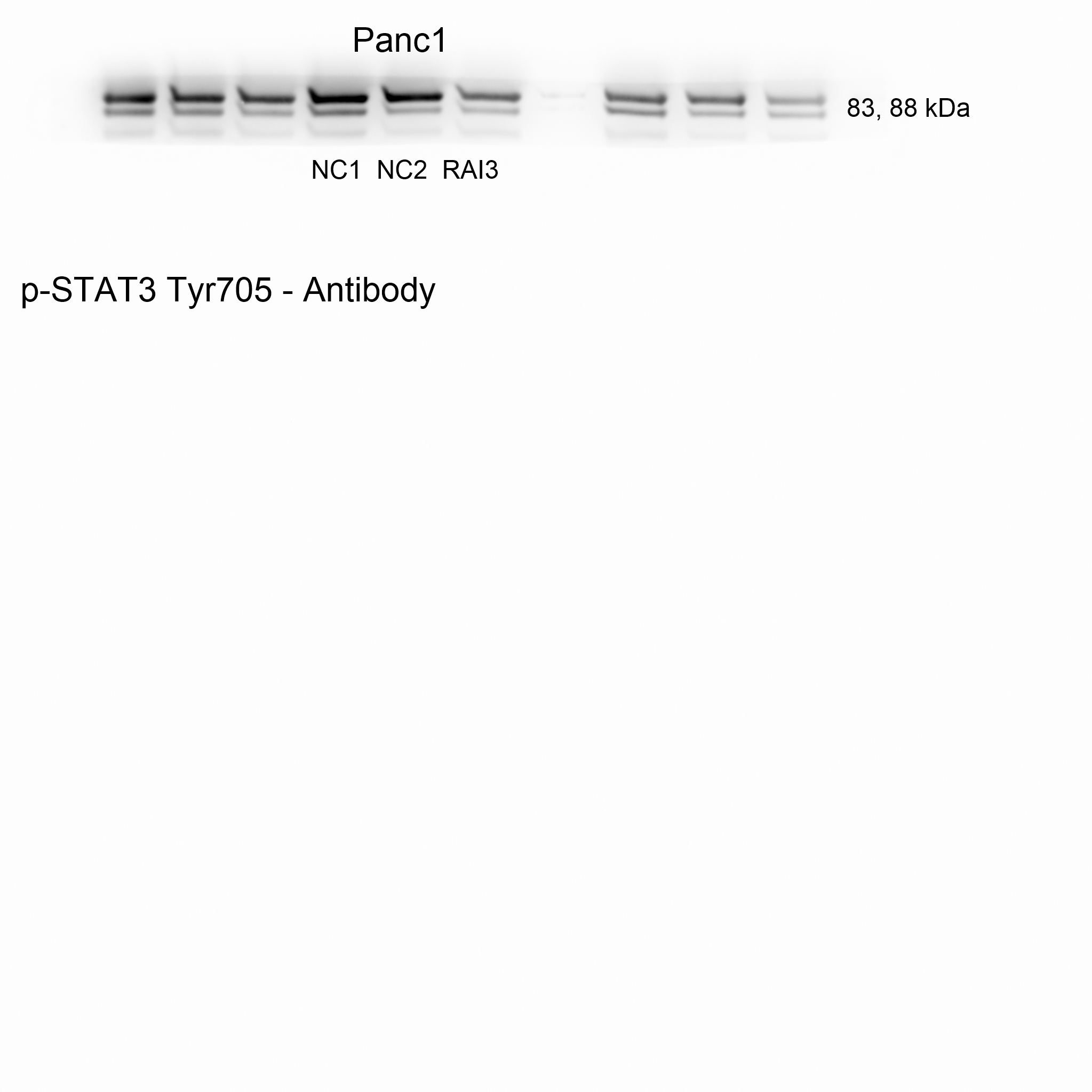

Supplement: S2 Fig — (ZIP) [file pone.0170390.s002.zip › Figure6_pSTAT3_antibody_Panc1.tif]

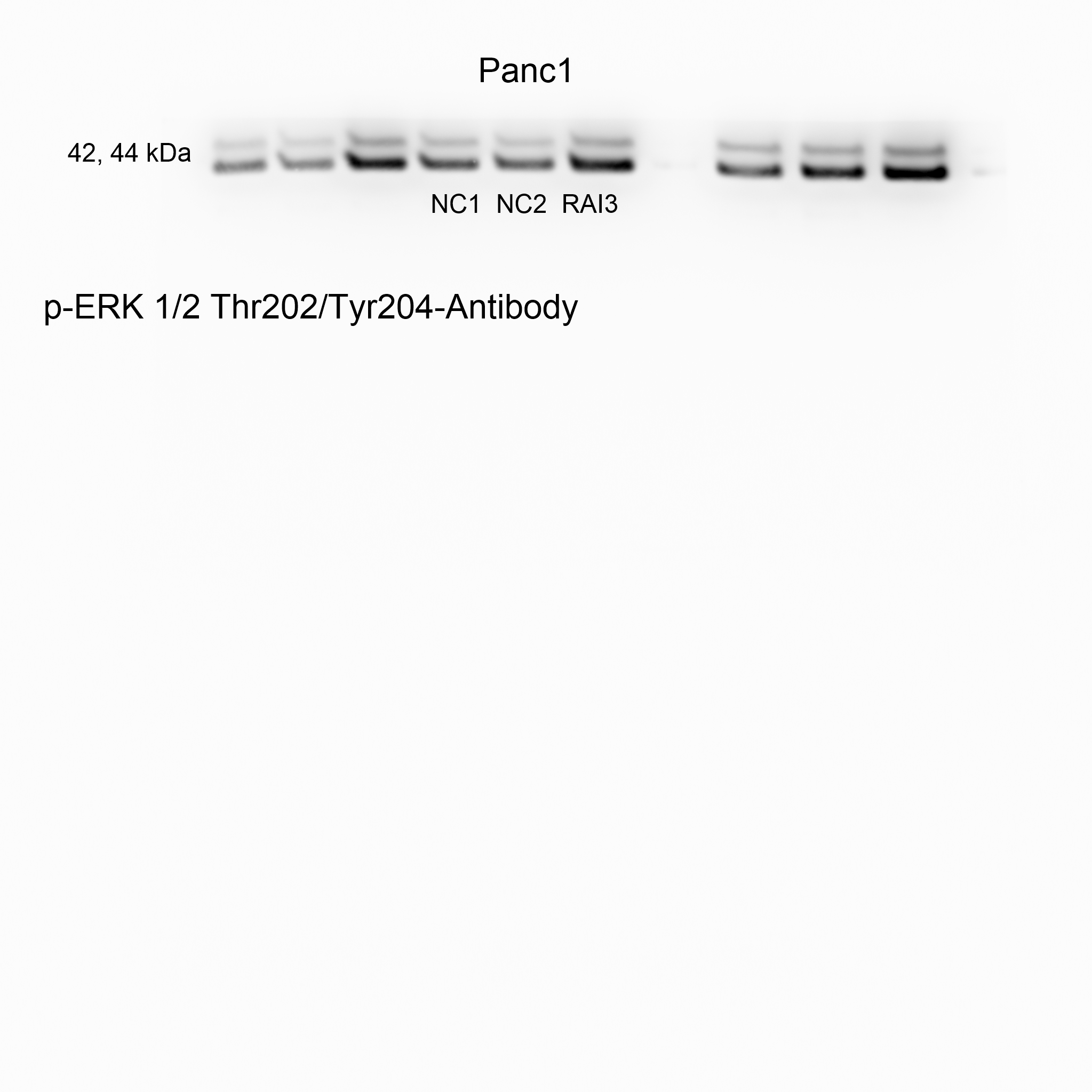

Supplement: S2 Fig — (ZIP) [file pone.0170390.s002.zip › Figure6_pERK_antibody_Panc1.tif]

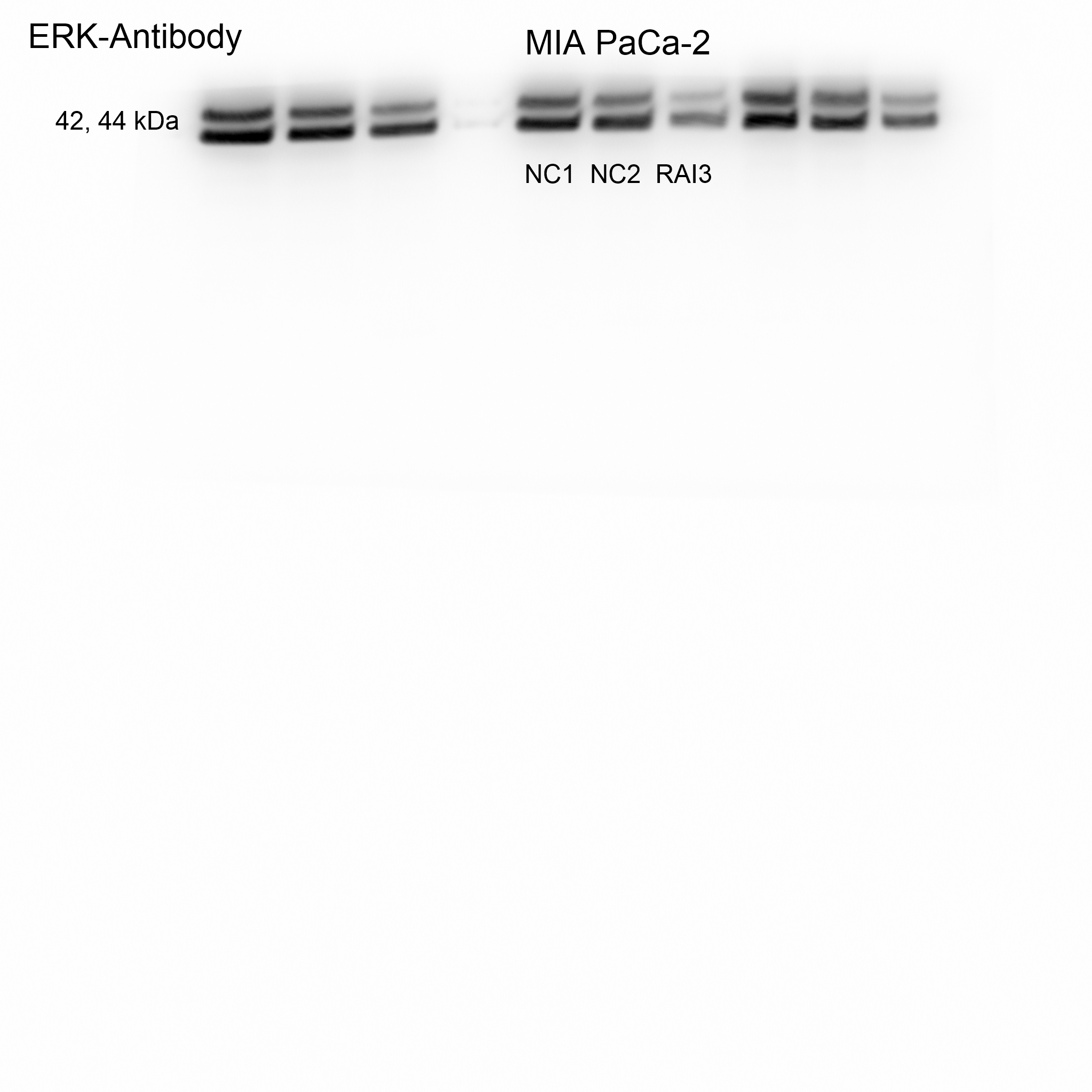

Supplement: S2 Fig — (ZIP) [file pone.0170390.s002.zip › Figure6_ERK_antibody_MIA PaCa-2.tif]

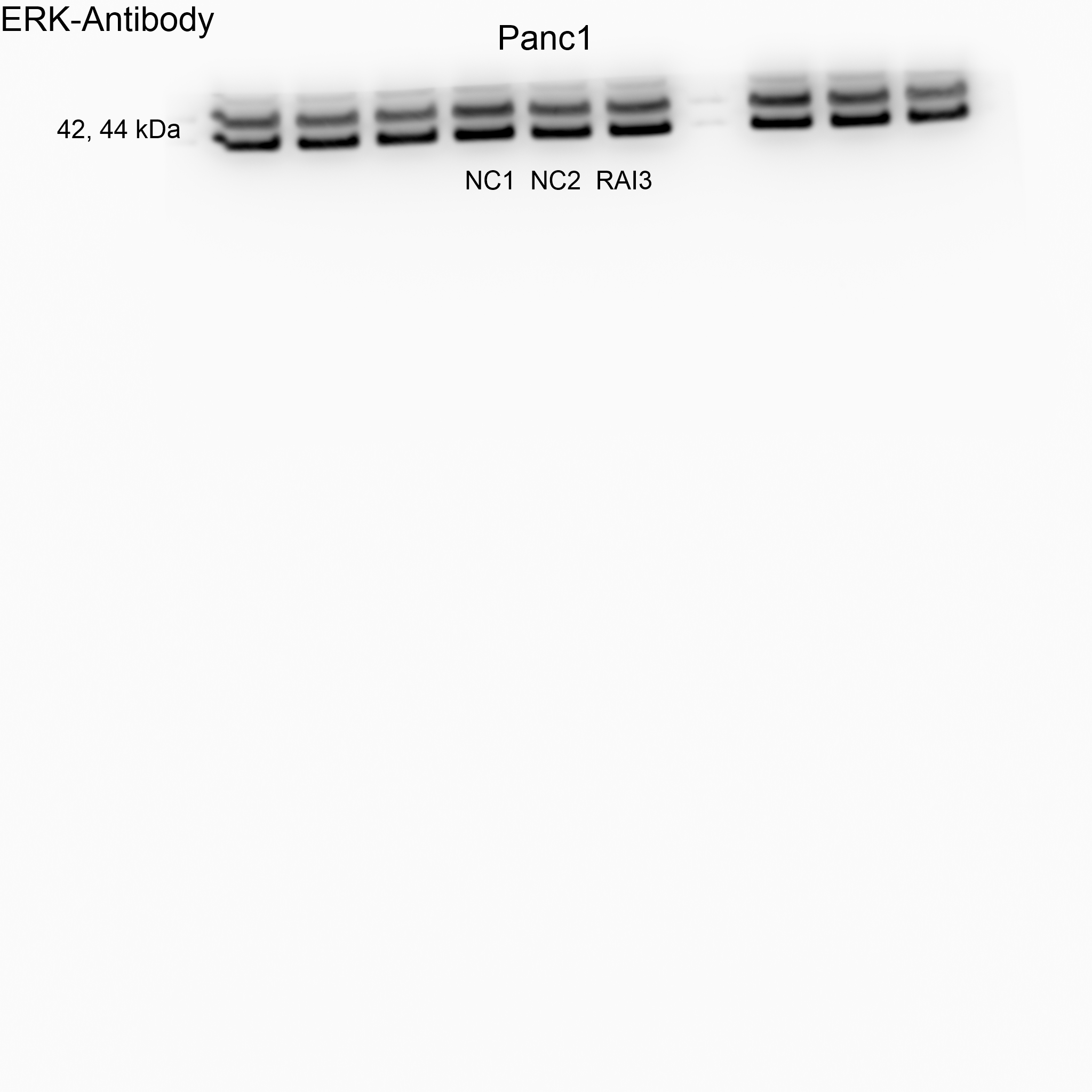

Supplement: S2 Fig — (ZIP) [file pone.0170390.s002.zip › Figure6_ERK_antibody_Panc1.tif]

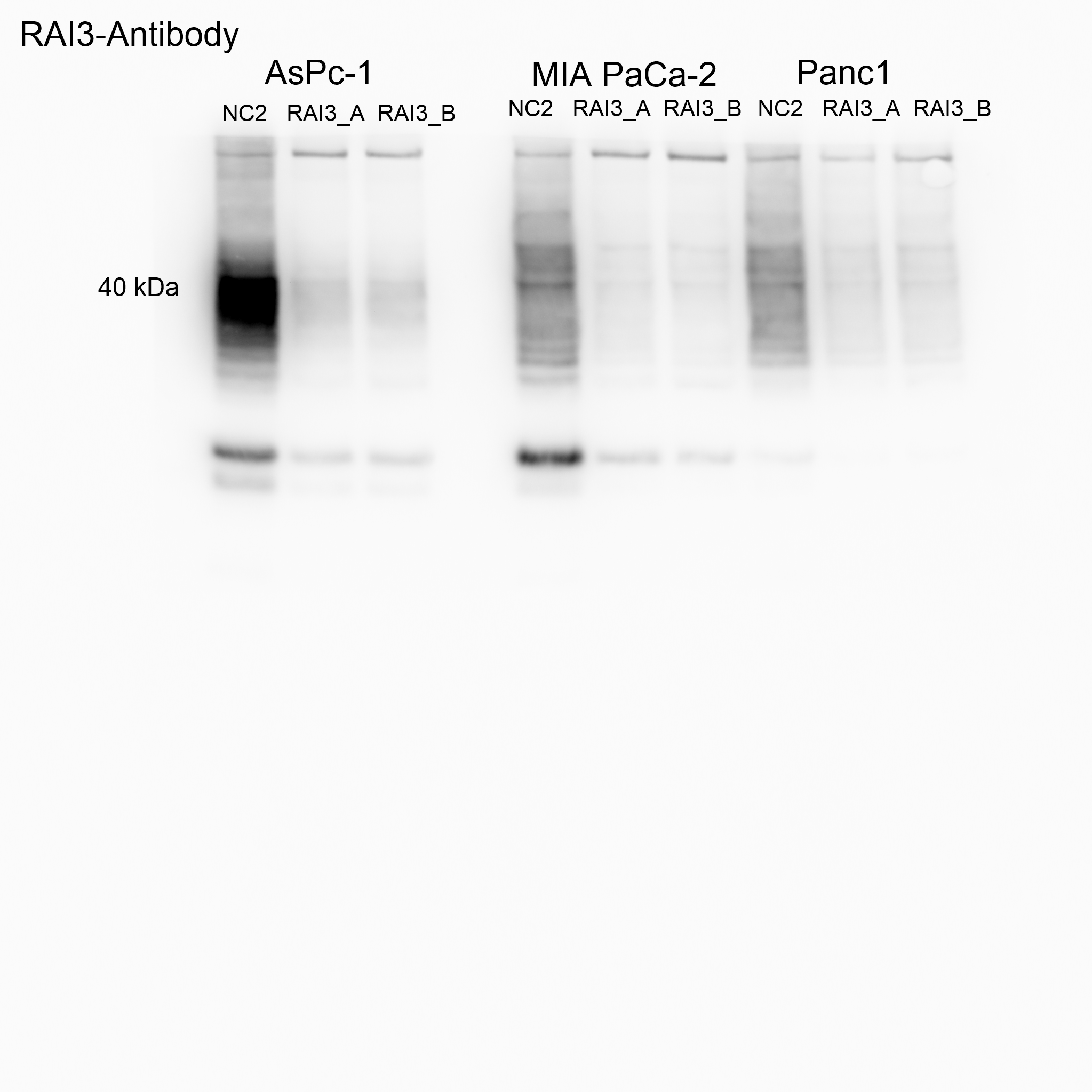

Supplement: S2 Fig — (ZIP) [file pone.0170390.s002.zip › Figure4B_RAI3_antibody.tif]

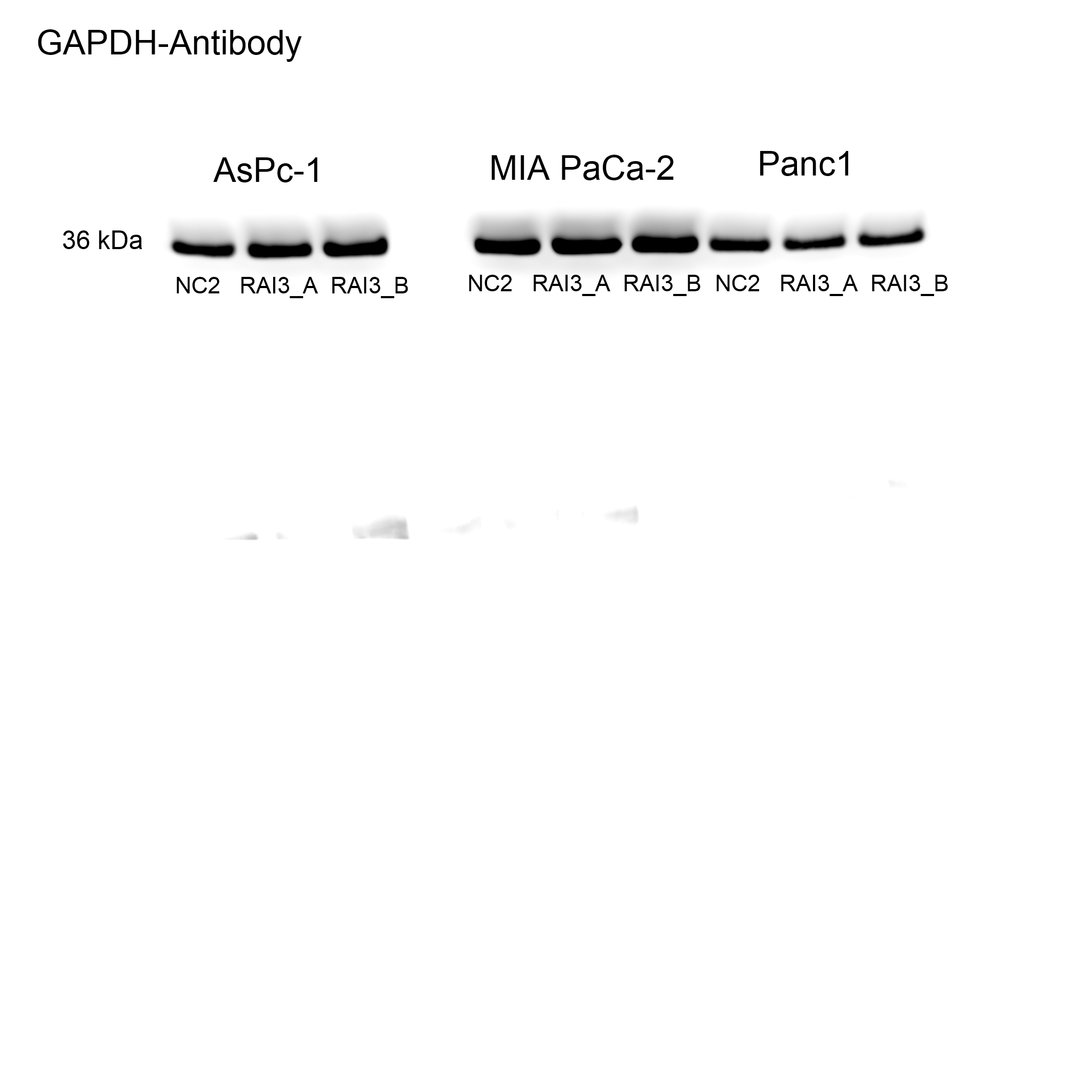

Supplement: S2 Fig — (ZIP) [file pone.0170390.s002.zip › Figure4B_GAPDH_antibody.tif]

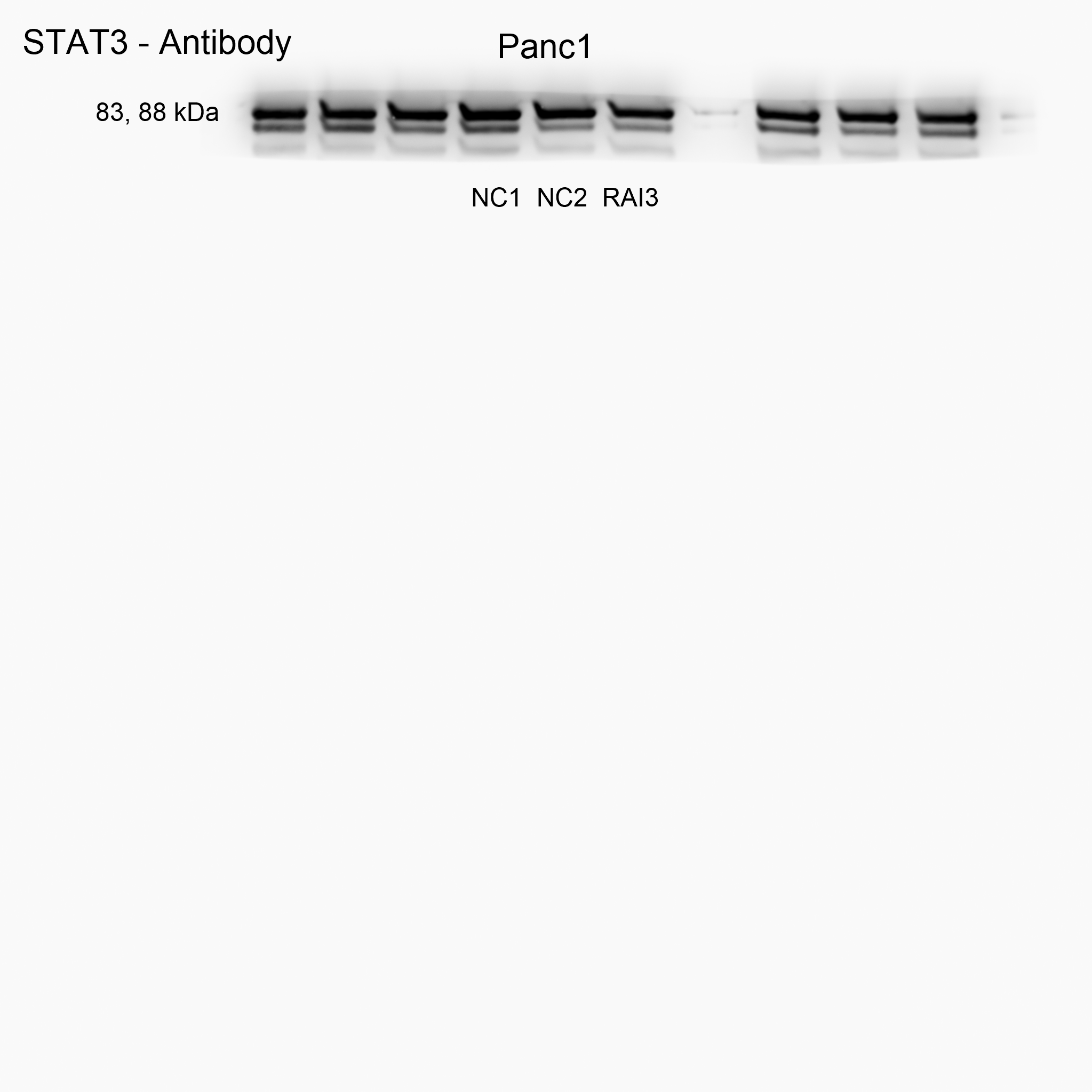

Supplement: S2 Fig — (ZIP) [file pone.0170390.s002.zip › Figure6_STAT3_antibody_Panc1.tif]

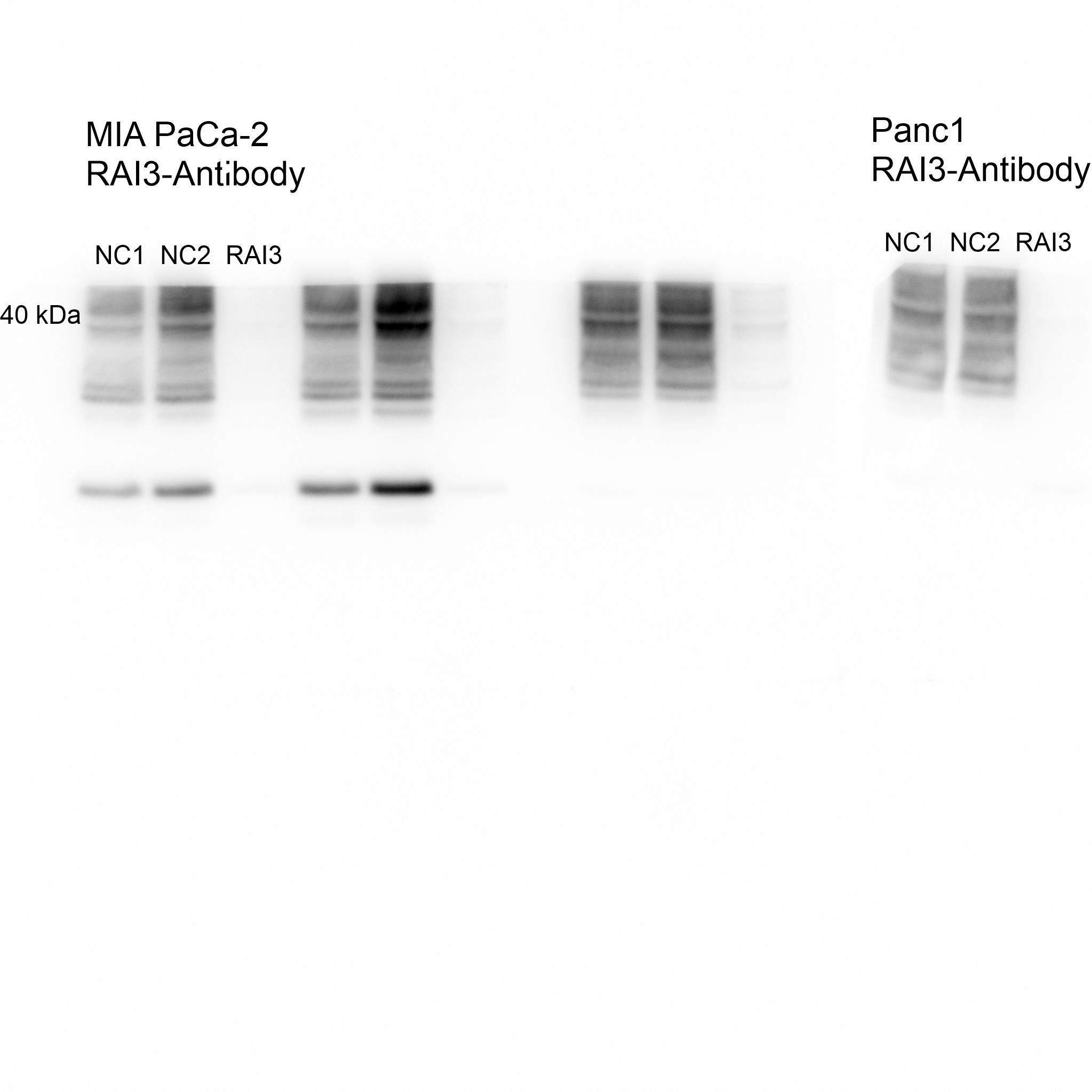

Supplement: S2 Fig — (ZIP) [file pone.0170390.s002.zip › Figure6_RAI3_antibody_MIA_Panc1.tif]

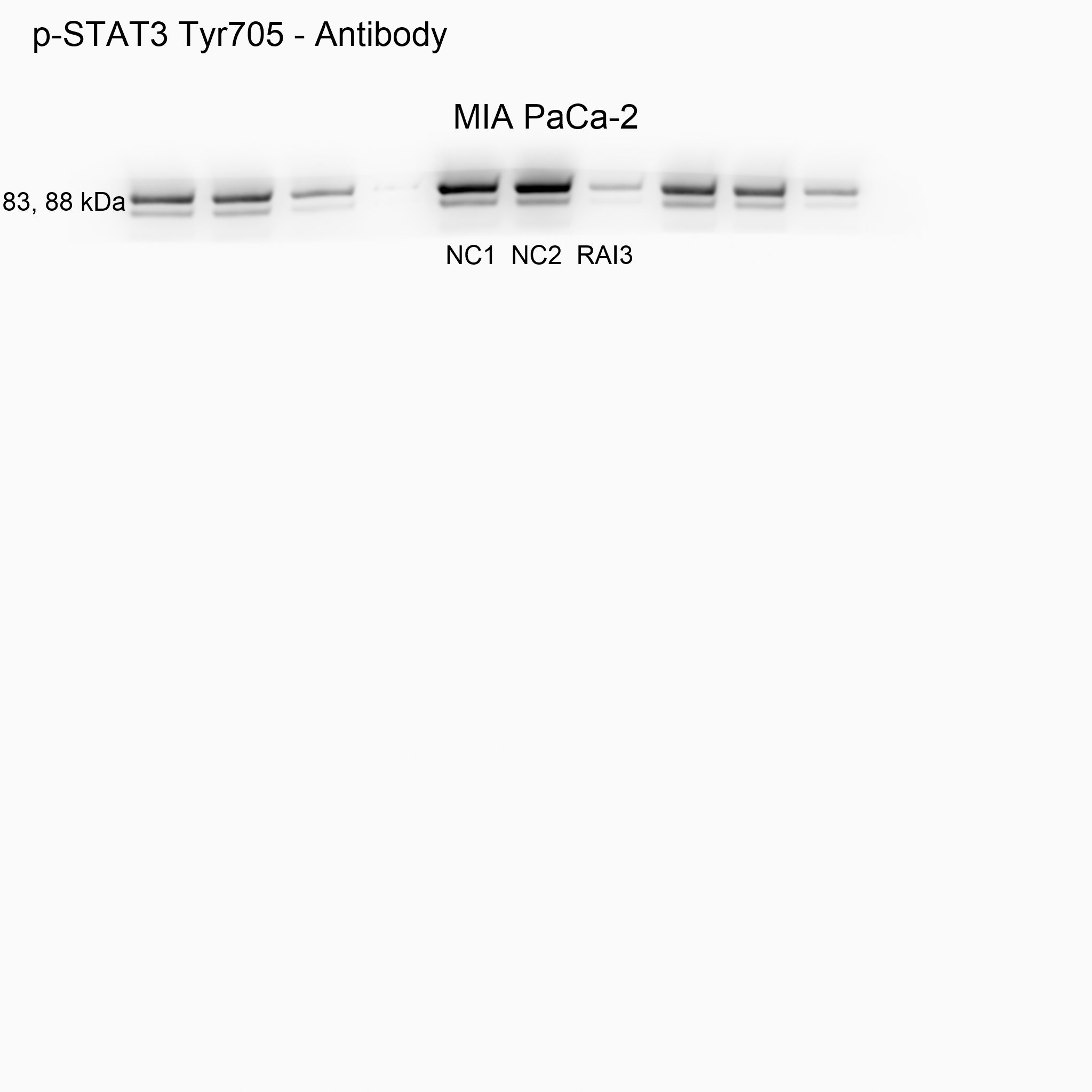

Supplement: S2 Fig — (ZIP) [file pone.0170390.s002.zip › Figure6_pSTAT3_antibody_MIA PaCa-2.tif]

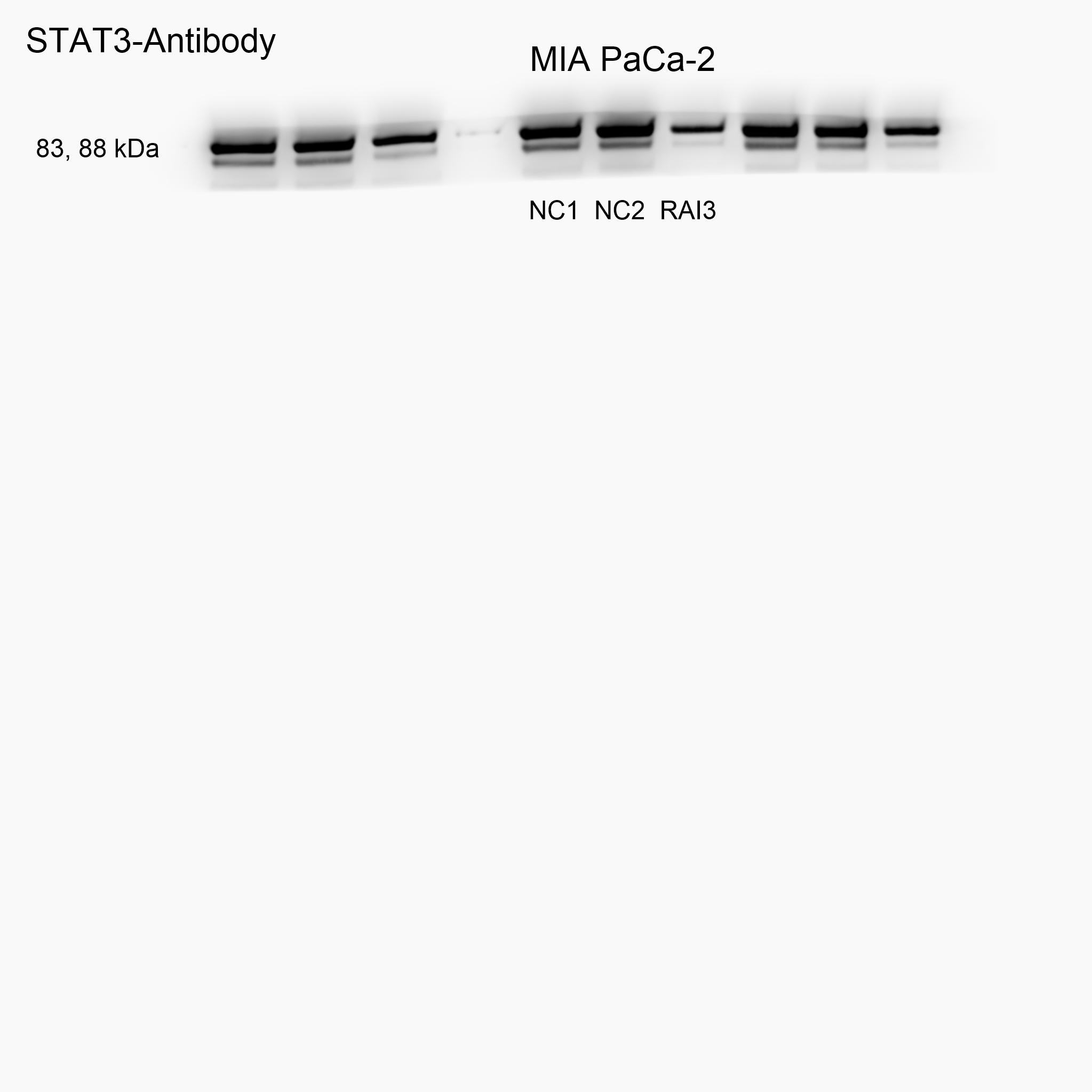

Supplement: S2 Fig — (ZIP) [file pone.0170390.s002.zip › Figure6_STAT3_antibody_MIA PaCa-2.tif]

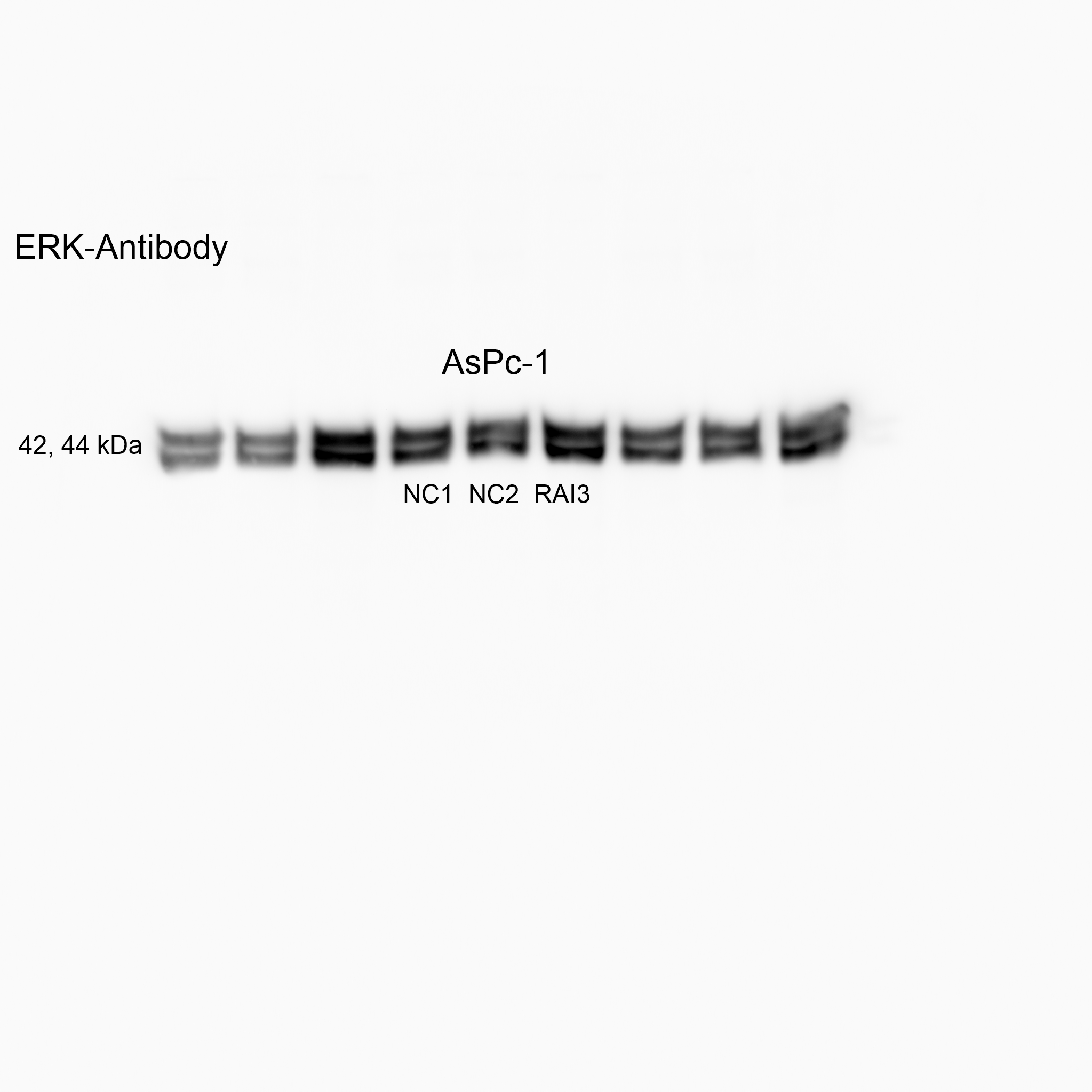

Supplement: S2 Fig — (ZIP) [file pone.0170390.s002.zip › Figure6_ERK_antibody_AsPc-1.tif]

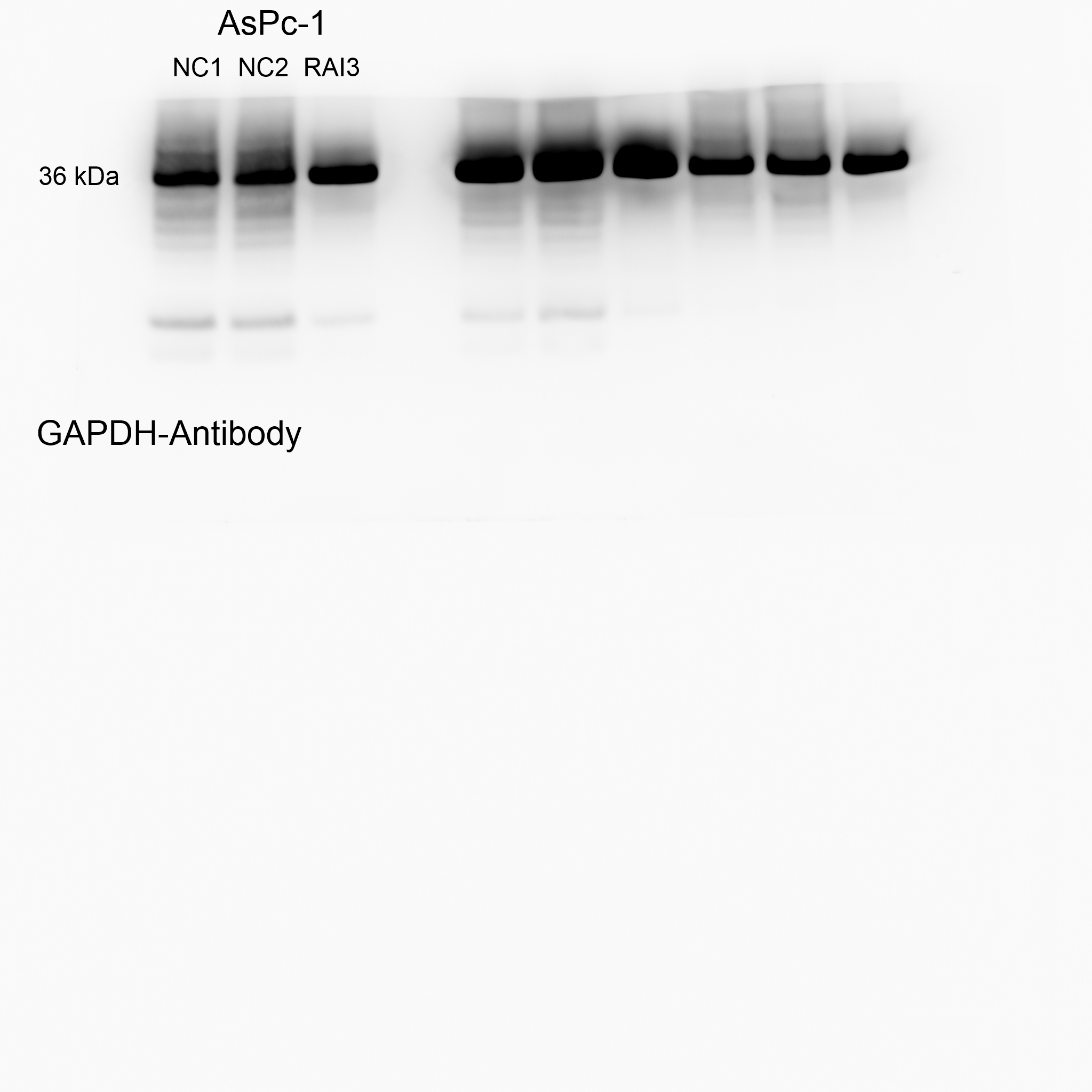

Supplement: S2 Fig — (ZIP) [file pone.0170390.s002.zip › Figure6_GAPDH_antibody_AsPc-1.tif]
